# Astaxanthin as a Potential Neuroprotective Agent for Neurological Diseases

**DOI:** 10.3390/md13095750

**Published:** 2015-09-11

**Authors:** Haijian Wu, Huanjiang Niu, Anwen Shao, Cheng Wu, Brandon J. Dixon, Jianmin Zhang, Shuxu Yang, Yirong Wang

**Affiliations:** 1 Department of Neurosurgery, Sir Run Run Shaw Hospital, School of Medicine, Zhejiang University, Hangzhou 310016, China; E-Mails: haijwu@sina.com (H.W.); huanjiangniu@163.com (H.N.); chengwusy@sina.com (C.W.); 2 Department of Neurosurgery, Second Affiliated Hospital, School of Medicine, Zhejiang University, Hangzhou 310009, China; E-Mails: awshao@sina.com (A.S.); zjmvip135@sina.com (J.Z.); 3 Department of Physiology and Pharmacology, School of Medicine, Loma Linda University, Loma Linda, CA, 92350, USA; E-Mail: Bjdixon@llu.edu

**Keywords:** astaxanthin, oxidative stress, inflammation, apoptosis, neuroprotection, neurological diseases

## Abstract

Neurological diseases, which consist of acute injuries and chronic neurodegeneration, are the leading causes of human death and disability. However, the pathophysiology of these diseases have not been fully elucidated, and effective treatments are still lacking. Astaxanthin, a member of the xanthophyll group, is a red-orange carotenoid with unique cell membrane actions and diverse biological activities. More importantly, there is evidence demonstrating that astaxanthin confers neuroprotective effects in experimental models of acute injuries, chronic neurodegenerative disorders, and neurological diseases. The beneficial effects of astaxanthin are linked to its oxidative, anti-inflammatory, and anti-apoptotic characteristics. In this review, we will focus on the neuroprotective properties of astaxanthin and explore the underlying mechanisms in the setting of neurological diseases.

## 1. Introduction

Neurological diseases, exemplified by acute injuries (e.g., stroke and traumatic brain injury) and chronic neurodegeneration (e.g., Alzheimer’s disease, Parkinson’s disease, and Huntington’s disease), are common causes of human death and disability [1,2]. Oxidative stress, inflammation, and apoptosis are some of the mechanisms involved in the pathogenesis of these diseases [3,4]. For example, highly insoluble amyloid beta peptide deposits and neurofibrillary tangles provide obvious stimuli for oxidative stress and inflammation in a brain with Alzheimer’s disease (AD), which significantly contributes to neuronal death in this disease [5,6,7]. In addition, there is evidence demonstrating that mitochondrial deficits, oxidative and nitrosative stress, accumulation and aggregation of aberrant or misfolded proteins (*i.e.*, α-synuclein), and dysfunction of ubiquitin-proteasome system represents the principal molecular events that commonly underlie the pathogenesis of familial and sporadic forms of Parkinson’s disease (PD) [8,9]. Additionally, highly polymorphic CAG tri-nucleotide repeat expansions in exon-1 of the *huntingtin* gene encodes an abnormally long poly-glutamine repeat, which is associated with Huntington’s disease (HD)-related brain pathology [10]. Poly-glutamine expansion causes huntingtin to aggregate and accumulate in the nucleus. This leads to abnormal interactions with other proteins, which results in intra-nuclear accumulation of mutant huntingtin and the formation of neuropil aggregates that may ultimately lead to neuronal cell death [11]. Therefore, multi-targeted pharmacological agents may be effective for the treatment of these devastating diseases.

Astaxanthin, a unique member of the xanthophylls, is a deep red-colored phytonutrient that can be synthesized by a microalgae called *Haematococcus pluvialis* [12]. Distinct from other members of the xanthophylls, astaxanthin has two hydroxyl groups [13]. Astaxanthin spans the bi-lipid layer and is long enough that the two hydroxyl groups jut into the fluid phase near the membrane, and that when electrons are extracted from these hyroxyl groups by free radicals, the molecule is resonance stabilized. As a consequence, these properties allow astaxanthin to do a lot in the body. For instance, astaxanthin can dramatically decrease the risk of cardiovascular disease [14]. A diet supplemented with astaxanthin (75 or 200 mg/kg body weight per day) for 8 weeks has been shown to improve endothelium-dependent vasodilatation in resistance vessels, reduce systolic blood pressure, and improve cardiovascular remodeling in spontaneously hypertensive rats [15]. In addition, astaxanthin (100 and 500 mg/100 g) for 60 days protects against serum protein oxidation in hyper-cholesterolemic rabbits [16]. Studies have also demonstrated that astaxanthin can easily cross the BBB to protect the brain from acute injury and chronic neurodegeneration [17,18]. The neuroprotective properties of this molecule involves anti-oxidation, anti-inflammation, and anti-apoptotis [19,20,21]. Thus, this review article will focus on the beneficial effects of astaxanthin and explore the underlying mechanisms observed in experimental models of neurological diseases. We also propose that further studies involving astaxanthin are needed, in order to evaluate its potential application in the treatment of neurological disorders.

## 2. Astaxanthin: Source, Biochemistry, Bioavailability, and Safety

Xanthophyll is a class of oxygen-containing carotenoid pigments whose biosynthesis in plants derives from the lycopene. Astaxanthin is a reddish pigment which belongs to the xanthophyll family [22]. This compound naturally exists in a wide variety of living organisms which includes microalgae, complex plants, and seafood [23]. The commercial form of astaxanthin is mainly synthesized from the algae *Haematococcus pluvialis* and the yeast *Phaffia rhodozyma*. As a member of the xanthophyll group, astaxanthin is closely related to other carotenoids such as β-carotene, lutein, and zeaxanthin. As a member of the xanthophyll group, astaxanthin is closely related to other carotenoids such as β-carotene, lutein, and zeaxanthin [24]. Similarly, they share many of the physiological and metabolic functions attributed to carotenoids [25]. Unlike β-carotene, astaxanthin does not have pro-vitamin A activity in the human body [26].

The molar mass of astaxanthin is 596.84 g/mol and the molecular formula is C_40_H_52_O_4_. It is a symmetric molecule consisting of two terminal rings joined by a short polyene ring [22]. The hydroxyl group at the end of the molecule enables it to esterify fatty acids to form mono-esters or di-esters [13]. Natural astaxanthin mainly exists in an esterified form, while the synthetic form is produced in a free form [27]. Astaxanthin also contains conjugated double bonds, giving this molecule strong anti-oxidant properties by donating electrons and reacting with free radicals to terminate free radical chain reactions within cells [25,28].

Astaxanthin has both lipophilic and hydrophilic properties, since it is fat-soluble and can be carried by fat molecules directly to tissues and organs that need it the most, like the brain, retina, and skeletal muscle [22]. Astaxanthin is first absorbed into enterocytes through passive diffusion and undergoes facilitated diffusion in the presence of lipids [29]. The unesterified forms are incorporated into chylomicrons and are transported into the liver via the lymphatic system [30]. The liver does not biochemically convert these molecules into vitamin A [31]. Instead, it is incorporated into lipoproteins that are transported into organs and tissues via the circulation [32].

Astaxanthin is safe to consume with food and contains no reports of side effects [33,34]. One randomized clinical trial found that 6 mg/day of astaxanthin can be safely consumed by healthy adults [35]. In addition, numerous human clinical trials have shown that the astaxanthin rich extract, *Haematococcus pluvialis*, is safe as well [36,37]. Hoffman-La Roche confirmed the safety of astaxanthin with acute, mutagenicity, teratogenicity, embryotoxicity, and reproductive toxicity tests [38]. In addition, the United States Food and Drug Administration approved the use of astaxanthin as a dietary supplement in 1999 [25].

## 3. Neuroprotective Properties of Astaxanthin in Neurological Diseases

There have been numerous studies concerning the beneficial effects of astaxanthin. Astaxanthin-mediated neuroprotection in experimental models of neurological disorders involves anti-oxidantion, anti-inflammation, and anti-apoptotic mechanisms [17,39]. The following sections will delve into these molecular mechanisms and their potential as treatments for neurological diseases.

### 3.1. Anti-Oxidant Effects

Oxidative stress is a key mediator in the pathology of neurological diseases [40,41,42]. Disturbance of the equilibrium status of pro-oxidant/anti-oxidant reactions in cells can lead to oxidative stress, which causes generation of reactive oxygen species (ROS) and free radicals [43]. When produced in excessive amounts, ROS like the superoxide anion radical (O_2_**^−^**) and its dismutation product, hydrogen peroxide (H_2_O_2_), are detrimental to metabolic functions [44,45]. The O_2_**^−^** radical can oxidize the [4Fe-4S] clusters of dehydratases, such as aconitase, causing inactivation and release of Fe^2+^ [46,47]. Thereafter, Fe^2+^ reacts with H_2_O_2_ to yield the potent oxidizing free radical species hydroxyl radical (OH). These substances further react with key organic substrates, such as DNA, proteins, and lipids, to disturb cell function and cause cell death [48]. It is worth mentioning that astaxanthin can act as a safeguard against oxidative damage through various mechanisms, by quenching of singlet oxygen, scavenging of radicals, inhibiting lipid peroxidation, and regulating gene expression related to oxidative stress [49,50,51,52]. For example, astaxanthin exerts beneficial effects against HgCl_2_-induced acute renal failure by preventing lipid and protein oxidation [53]. In an *in vivo* murine model, astaxanthin administration prevented *N*-Methyl-d-aspartate (NMDA)-triggered retinal damage, which is associated with decreasing lipid peroxidation and oxidative DNA damage [54]. Astaxanthin treatment ameliorates cyclophosphamide-induced oxidative stress and the subsequent DNA damage in rat hepatocytes [55]. The protective effect of this molecule is attributed to the activation of nuclear erythroid 2-related factor 2 (Nrf2) antioxidant response element (ARE) pathway, which eventually facilitates Nrf2-dependent gene expression of heme oxygenase-1 (HO-1) and NAD(P)H: quinine oxidoreductase-1 (NQO-1) [55]. In the human retinal pigment epithelial (RPE) cell line ARPE-19, astaxanthin inhibited the intracellular production of ROS and prevented H_2_O_2_-induced decrease in retinal pigment epithelial cell viability [56]. Astaxanthin also increased the nuclear translocation of Nrf2 and enhanced the expression of phase II anti-oxidant enzymes through the activation of the phosphoinositide 3-kinase (PI3K)/Akt pathway, which eventually provided protection against H_2_O_2_-induced oxidative stress in ARPE-19 cells [56].

**Figure 1:**
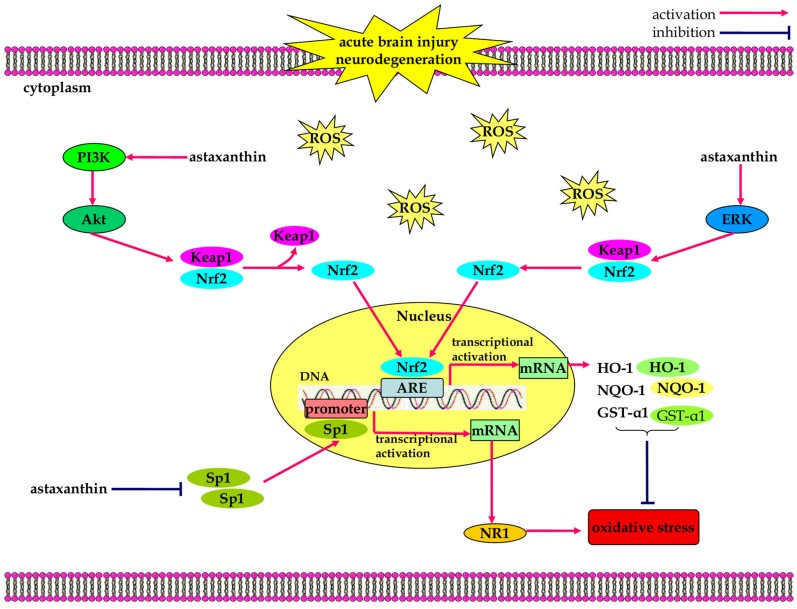
The anti-oxidative effects of astaxanthin in neurological diseases. Astaxanthin facilitates the dissociation and nuclear translocation of nuclear erythroid 2-related factor (Nrf2), through activation of the PI3K/Akt and ERK signaling pathways, which contributes to increased expression of Nrf2-regulated enzymes like HO-1, NQO-1, and GST-α1 that resist oxidative stress. In addition, astaxanthin negatively regulates Sp1/NR1 signaling pathway, alleviating the production of intracellular ROS and oxidative stress.

The anti-oxidative effects of astaxanthin have also been investigated in experimental models of acute neurological conditions (Figure 1). Lee *et al.* reported that astaxanthin provides neuroprotective effects against oxidative stress induced by oxygen-glucose deprivation in SH-SY5Y cells and 10-min global cerebral ischemia in rats [57]. In a murine model of ischemic stroke, pre-treatment with astaxanthin decreased ROS production and alleviated lipid peroxidation in the ipsilateral brain of rats subjected to middle cerebral artery occlusion (MCAO) [17]. Simultaneously, astaxanthin reduced cerebral infarction and promoted locomotor function recovery following MCAO [17]. Zhang *et al.*, found that administration of astaxanthin had the potential of alleviating early brain injury (EBI) after subarachnoid hemorrhage (SAH) through its anti-oxidative properties [19]. Treatment with astaxanthin is believed to confer protective effects by restoring endogenous anti-oxidant enzymes of glutathione (GSH) and superoxide dismutase (SOD) following SAH [19]. Wu *et al.* reported that post-SAH treatment of astaxanthin facilitated the Nrf2-ARE pathway and ameliorated EBI in a prechiasmatic cistern model of SAH [58]. Astaxanthin activated the Nrf2-ARE signaling pathway to up-regulate the expression of Nrf2-regulated enzymes like HO-1, NQO-1 and glutathione-*S*-transferase-α1 (GST-α1) to resist oxidative stress [58].

Astaxanthin also plays a role in preventing the development of chronic neurodegeneration. It boosted the expression of HO-1 and protected neurons against Aβ-induced cytotoxicity [59,60]. Astaxanthin-stimulated activation of extracellular regulated protein kinase (ERK) signaling pathway facilitated the dissociation of Nrf2 from Keap1, promoting the nuclear translocation and DNA-binding activity of Nrf2 leading to up-regulation of HO-1 expression and protection against Aβ-induced neurotoxicity [59]. In a cellular model of PD, astaxanthin reduced the generation of intracellular ROS and provided cytoprotective effects against 1-methyl-4-phenylpyridinium (MPP^+^)-induced cytotoxicity [61]. In addition, astaxanthin enhanced HO-1 expression and limited NADPH oxidase 2 (NOX2)-mediated oxidative damage in MPP^+^-treated PC12 cells [62]. Astaxanthin antagonized MPP^+^-induced oxidative stress through the regulation of specificity protein 1 (Sp1) and NMDA receptor subunit 1 (NR1) signaling pathway [63]. Pre-treatment with astaxanthin markedly inhibited the up-regulation and nuclear transfer of Sp1, thereby alleviated MPP^+^-induced production of intracellular ROS and cytotoxicity in PC12 cells [63]. Thus, astaxanthin provides protection against oxidative attacks in experimental neurological diseases.

### 3.2. Anti-Inflammatory Effects

**Figure 2:**
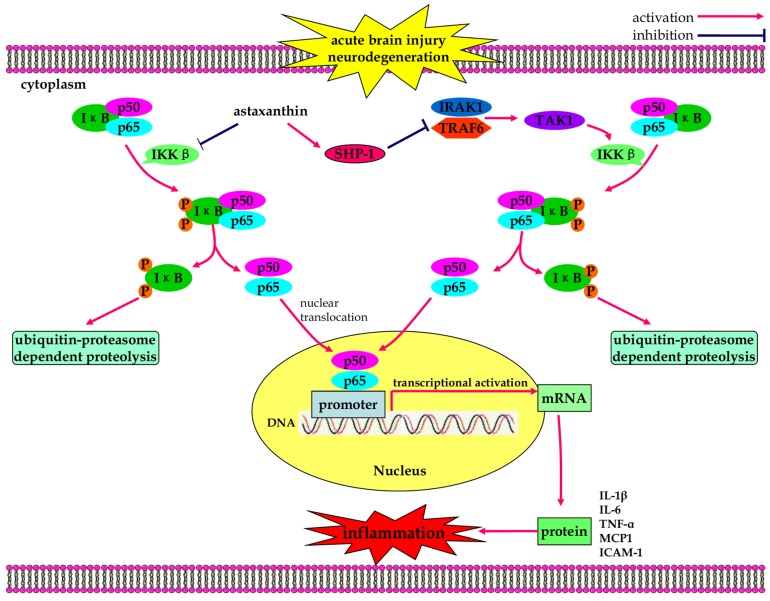
The anti-inflammatory effects of astaxanthin in neurological diseases. Through suppression of IκB-α degradation and NF-κB nuclear translocation, astaxanthin inhibits the expression of inflammatory molecules IL-6, ICAM-1, and MCP1. Astaxanthin also suppresses nuclear expression of NF-κB and reduces downstream production of pro-inflammatory cytokines by restoring physiological levels of SHP-1.

Inflammation is defined as series of complex immune responses that biologically occurs as a reaction to injuries of the body. It functions as a host defense mechanism to clear out damaged tissue from the original insult and initiates the tissue repair process [64]. However, excessive or uncontrolled inflammation is detrimental to the host and can cause damage to the host’s cells and tissues [65]. In the central nervous system (CNS), inflammation has a critical role in both acute conditions (*i.e.*, stroke and traumatic injury) and chronic neurodegenerative conditions (e.g., AD, PD, and HD) [66]. Interestingly, astaxanthin exhibits anti-inflammatory effects in lipopolysaccharide-induced uveitis by directly blocking the activity of inducible nitric oxide synthase (NOS) (Figure 2) [67]. In addition, astaxanthin suppressed gene expression of inflammatory mediators (*i.e.*, TNF-α and IL-1β) and alleviated endotoxin-induced uveitis by blocking the NF-κB-dependent signaling pathway [68]. Under normal conditions NF-κB, a heterodimer composed of p50 and p65 subunits, interacts with inhibitor of NF-κB (IκB) and remains inactive in the cytosol [69]. Upon stimulation, IκB undergoes phosphorylation by IκB kinase β (IKKβ) and is degraded via the ubiquitin proteasome pathway [70,71]. Dissociation of IκB from the p50/p65 heterodimer exposes the nuclear localization signal on NF-κB, which subsequently leads to the translocation of NF-κB (p65) into the nucleus to regulate the transcription of inflammatory genes [72]. Astaxanthin treatment effectively alleviated NF-κB-related inflammation in the liver of mice subjected to a high fructose and high fat diet by suppressing IKKβ phosphorylation and nuclear translocation of NF-κB (p65) subunit [73]. Astaxanthin also suppressed ROS-induced nuclear expression of NF-κB (p65) and reduced the downstream production of pro-inflammatory cytokines (*i.e.*, IL-1β, IL-6 and TNF-α) in U937 mononuclear cells by restoring the physiological levels of protein tyrosine phosphatase-1 (SHP-1) [74]. In a mouse model of experimental choroidal neovascularization, Izumi-Nagai demonstrated that astaxanthin treatment led to significant inhibition of macrophage infiltration into choroidal neovascularization [75]. Furthermore, astaxanthin suppressed IκB-α degradation and NF-κB nuclear translocation, resulting in subsequent down-regulation of inflammatory molecules (*i.e.*, IL-6, vascular endothelial growth factor (VEGF), intercellular adhesion molecule-1 (ICAM-1), and monocyte chemotactic protein 1 (MCP1) [75]. Astaxanthin also decreased gastric inflammation in mice infected with *Helicobacter pylori*, shifting the T-lymphocyte response from a Th1 response to a Th1/Th2 response [76]. Additionally, astaxanthin decreased nitric oxide (NO) production and inducible nitric oxide synthase (iNOS) activity in macrophages, resulting in inhibition of cyclooxygenase and down-regulation of prostaglandin E2 (PGE2) and TNF-α in mice [67]. Dietary administration of astaxanthin significantly suppressed aberrant NF-κB activation in colonic mucosa, lowering gene expressions of IL-1β, IL-6, and COX-2, which contributes to attenuation of dextran sulfate sodium (DSS)-induced colitis [77]. Lee and colleagues discovered that astaxanthin prevented inflammatory processes by suppressing the activation of NF-κB signaling and the production of pro-inflammatory cytokines (e.g., TNF-α and IL-1β) using both *in vitro* and *in vivo* models [78]. In human keratinocytes, Terazawa *et al.* demonstrated that astaxanthin interrupts the auto-phosphorylation and self-activation of mitogen- and stress-activated protein kinase-1 (MSK1), which results in decreased phosphorylation of NF-κB (p65) and deficiency of NF-κB DNA binding activity [79]. As a consequence, UVB-induced expression and secretion of PGE2 and IL-8 were down-regulated in these human keratinocytes [79].

In a prechiasmatic cistern SAH model, astaxanthin provides neuroprotection against EBI through suppression of cerebral inflammation [20]. Post-treatment with astaxanthin after SAH reduced neutrophil infiltration, suppressing the activity of NF-κB, decreasing the protein and mRNA levels of inflammatory mediators IL-1β, TNF-α, and ICAM-1, and dramatically reversed brain inflammation [20]. As a result secondary brain injury cascades, neuronal degeneration, BBB disruption, cerebral edema, and neurological dysfunction, were all alleviated after astaxanthin administration [20]. However, there is still a lack of research documenting the anti-inflammatory effects of astaxanthin on the treatment of neurological disorders. Several studies have reported that astaxanthin can enhance both humoral and cell-mediated immune responses [26,80,81,82,83]. Dietary supplement of astaxanthin can stimulate T cell and B cell mitogen-induced lymphocyte proliferation, increase the cytotoxic activity of natural killer cell, and enhance IFN-γ and IL-6 production in young healthy adult female human subjects [84]. Additionally, Balietti *et al.* showed gender-related differences in the anti-inflammatory effects of astaxanthin on the aging rat brain [85]. However, it is still unknown if this molecule exerts different anti-inflammatory effects in female and male brains under pathological conditions. Therefore, there is a need for future studies elucidating the inflammatory regulation mechanisms of astaxanthin.

### 3.3. Anti-Apoptotic Effects

Apoptosis is a highly sophisticated energy-dependent process of programmed cell death [86]. Morphologically, it is characterized by shrinkage of the cell, membrane blebbing, nuclear fragmentation and chromatin condensation [87]. Under normal physiological conditions, apoptosis is vital for embryonic development and tissue homeostasis [88]. Under pathological conditions, uncontrolled apoptosis is harmful and contributes to the pathogenesis of a variety of human diseases including neurological disorders [89]. Kim *et al.* demonstrated that astaxanthin provided protection against H_2_O_2_-mediated apoptosis in a mouse neural progenitor cell culture model [90]. Astaxanthin is believed to inhibit H_2_O_2_-mediated apoptotic cell death by maintaining mitochondria integrity, reducing cytochrome c release from the mitochondria, and inhibiting caspase activation in astaxanthin pre-treated cells through the modulation of p38 and mitogen-activated protein kinase kinase (MEK) signaling pathways in neural progenitor cells from mice [90]. Dong *et al.* reported that astaxanthin significantly reduced apoptotic death of retinal ganglion cells and alleviated diabetic retinopathy by oxidative stress inhibition [91]. In addition, astaxanthin administration increased Akt, enhanced Bad phosphorylation, and down-regulated the activation of downstream pro-apoptotic proteins (e.g., cytochrome c and caspase-3/9), leading to the amelioration of mitochondrial-related apoptosis and the attenuation of early acute kidney injury following severe burns [92].

**Figure 3:**
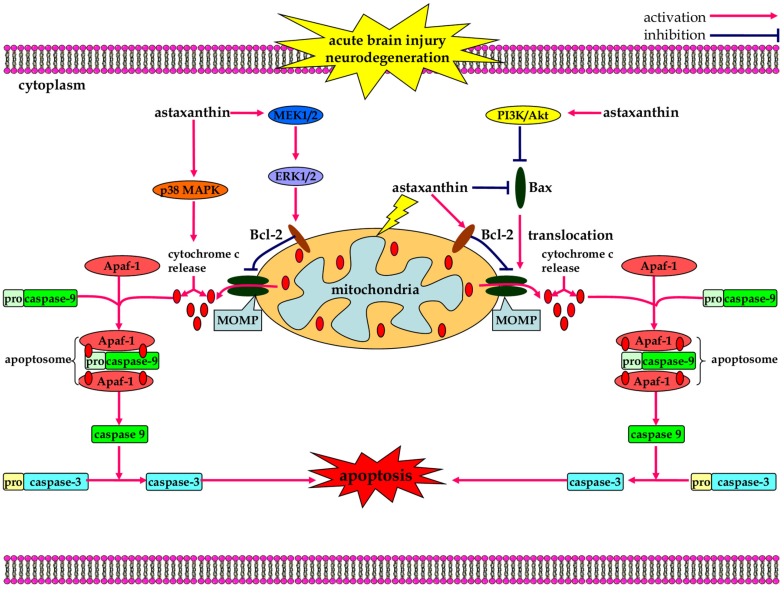
The anti-apoptotic effects of astaxanthin in neurological diseases. Astaxanthin induces the activation of PI3K/Akt survival pathway, promoting the phosphorylation-dependent inactivation of Bad, which leads to a decrease in caspase-dependent neuronal apoptosis. Astaxanthin also maintains mitochondria integrity through modulation of p38 and MEK signaling pathways, which reduces cytochrome c release and inhibits caspase-dependent apoptotic cell death.

Astaxanthin exerts a protective effect against neuronal apoptosis in the setting of neurological diseases as well (Figure 3). For example, astaxanthin mediated the activation of the PI3K/Akt survival pathway, promoted the phosphorylation-dependent inactivation of Bad, and decreased caspase-dependent neuronal apoptosis after SAH [21]. As a result, secondary brain injury in the early period of SAH, BBB disruption, cerebral edema, neurological deficits were all alleviated after treatment with astaxanthin [21]. Intra-cerebroventricular administration of astaxanthin antagonized ischemia/reperfusion-induced translocation of cytochrome c from the mitochondria to the cytoplasm, and prevented apoptosis in a transient MCAO model of ischemic stroke [17]. Lu *et al.* In addition, reported similar findings demonstrating that astaxanthin exhibits noticeable neuroprotection against cerebral ischemia-reperfusion insults through its anti-apoptotic actions [93]. In addition, pre-treatment with astaxanthin also significantly restored the mitochondrial membrane potential, prevented H_2_O_2_-induced neuronal apoptosis, decreased cerebral infarct volume, and improved neurological function after MCAO [93].

In an *in vitro* model of PD, Ikeda *et al.* demonstrated that astaxanthin attenuates 6-hydroxydopamine (6-OHDA)-induced apoptosis in human neuroblastoma SH-SY5Y cells [94]. Pre-treatment with astaxanthin significantly inhibits ROS generation and subsequent phosphorylation of p38 MAPK, ameliorates mitochondrial dysfunction, increases ΔΨm, reduces cytochrome c release, caspase activation, and rescues the cell from 6-OHDA-induced apoptosis [94,95]. Lee and coworkers found that astaxanthin treatment prevents MPP^+^-induced up-regulation of Bax and down-regulation of Bcl-2, alleviating ΔΨm collapse in SH-SY5Y cells and protects the neuron against MPP^+^-induced mitochondrial damage and apoptosis [96]. Liu *et al.* demonstrated that astaxanthin has protective effects on 6-OHDA-induced cellular toxicity and apoptotic death of dopaminergic SH-SY5Y cells by inhibiting intracellular ROS generation, the decrease of mitochondrial membrane potential, the release of mitochondrial cytochrome c [97].

Intrestingly, it has been shown that astaxanthin induces cancer cell apoptosis through a mitochondrial-dependent pathway [98]. Astaxanthin mediates the inhibition of the Janus kinase 1 (JAK1)/STAT3 (signal transducer and activator of transcription 3) signaling pathway in hepatocellular carcinoma CBRH-7919 cells which down-regulates the anti-apoptotic gene expression of Bcl-2 and Bcl-xl, while also enhancing the pro-apoptotic gene expression of Bax resulting in apoptosis [99]. Another study also reported that astaxanthin induces caspase-mediated mitochondrial apoptosis by down-regulating the expression of anti-apoptotic Bcl-2 and survivin while up-regulating pro-apoptotic Bax and Bad [100]. It has also been reported that astaxanthin can induce the intrinsic apoptotic pathway in a hamster model of oral cancer through the inactivation of ERK/MAPK and PI3K/Akt cascades which leads to the inhibition of NF-κB and Wnt/β-catenin [100]. Thus, depending on the pathological condition, astaxanthin may exert either anti-apoptotic or pro-apoptotic effects.

## 4. Conclusions and Perspective

Astaxanthin confers multiple neuroprotective effects in various experimental models of neurological diseases, which includes both acute injuries and chronic neurodegenerative disorders. The protective effects of astaxanthin are associated with its anti-oxidative, anti-inflammatory, anti-apoptotic effects. Astaxanthin is a safe nutrient, with no toxic effects when it is consumed with food. Furthermore, as a fat-soluble compound, astaxanthin is able to effectively pass through the BBB. Therefore, astaxanthin is an excellent candidate for treating neurological diseases. It is essential that there continues to be further evaluations of the protective properties and underlying mechanisms of astaxanthin, which may eventually lead to astaxanthin becoming a novel neuroprotective agent.

Although the neuroprotective effects of astaxanthin have been examined in several experimental models of neurological disorders, there is a lack of research in some areas. Future studies should focus on the pharmaceutical potential and effects of astaxanthin esters in the treatment of neurological disorders, especially since astaxanthin diesters can be easily absorbed into the metabolism and may increase biological activity more effectively than its free form [13]. Furthermore, it is important to note that a lot of the current data concerning astaxanthin-mediated neuroprotection mainly comes from ischemic stroke, SAH, AD, and PD. There is minimal evidence available regarding other neurological diseases such as traumatic brain injury, intracerebral hemorrhage, and HD. Therefore, future investigations should include these neurological disease models. The therapeutic time window, reliability of drug administration routes, and the optimal dosages of astaxanthin are other areas that need to be explored and determined. Most importantly, the development of clinical trials to assess astaxanthin as treatment of neurological diseases is warranted since there are a number of promising general safety results, neurological experimental model studies, and clinical trials in other diseases.
